# The therapeutic potential of amifostine on cyclophosphamide-induced testicular dysfunction in rats: An experimental study

**DOI:** 10.18502/ijrm.v17i4.4549

**Published:** 2019-06-13

**Authors:** Coskun Kaya, Ali Barbaros Baseskioglu, Semra Yigitaslan, Fikriye Yasemin Ozatik, Orhan Ozatik, Sema Uslu

**Affiliations:** ^1^Department of Urology, Eskisehir City Hospital, Eskisehir, Turkey.; ^2^Department of Urology, Eskisehir Acibadem Hospital, Eskisehir, Turkey.; ^3^Department of Pharmacology, Eskisehir Osmangazi University, Eskisehir, Turkey.; ^4^Department of Pharmacology, Kutahya Health Sciences University, Kutahya, Turkey.; ^5^Department of Histology and Embryology, Kutahya Health Sciences University, Kutahya, Turkey.; ^6^Department of Biochemistry, Eskisehir Osmangazi University, Eskisehir, Turkey.

**Keywords:** *Amifostine*, * Cyclophosphamide*, * Rat*, * Testis*

## Abstract

**Background:**

Cyclophosphamide (CP) is a well-known alkylating anticancer agent used in the treatment of various malignant and non-malignant tumors. CP may also cause a variety of adverse effects, including reproductive toxicity. Amifostine is known as a cytoprotective drug having antioxidant properties.

**Objective:**

To evaluate the possible beneficial effects of amifostine on testicular toxicity induced by CP in rats.

**Materials and Methods:**

A total of 35 Sprague-Dawley rats were used in this experimental study. The CP group animals received a single dose of 200 mg/kg CP on Day 8 by intraperitoneal injection and were left untreated for the following seven days. The two remaining groups of animals were treated with 200 mg/kg/day amifostine (AMF 200) and 400 mg/kg/day amifostine (AMF 400) for seven days prior to and following a single intraperitoneal injection of CP. Morphometrical analysis and histological examination of testicular tissue were performed. Serum testosterone, luteinizing hormone, and follicle-stimulating hormone levels were measured in serum using commercial ELISA kits. The epidydimal sperm count was determined.

**Results:**

The tubular epithelial height in the testis was significantly higher in the AMF400 group compared to other groups (p < 0.001). Animals in the AMF400 group showed minimal debris in the tubules, no Sertoli cell damage, and the Johnsen scores were slightly higher in the AMF400 group. The epididymal sperm count was significantly lower in the CP-administered animals compared to the control animals and was significantly higher in the AMF200 and AMF400 groups compared to the CP group (p = 0.006, and p = 0.019 respectively).

**Conclusion:**

Amifostine, at a dose of 400 mg/kg, may have a protective effect on testicular damage induced by CP in rats.

## 1. Introduction

Cyclophosphamide (CP) is a well-known alkylating anticancer agent used in the treatment of various malignant and non-malignant tumors (1). It is also used as an immunosuppressive agent to prevent organ transplant rejection and to treat several autoimmune diseases. Although it is widely used in clinical practice, CP may also cause a variety of adverse effects, including reproductive toxicity, which is particularly significant in younger patients (2, 3). Although the exact mechanism of testicular toxicity induced by CP is unclear, there is clear evidence that CP disrupts the redox balance of tissues and induces oxidative stress (3–5).

The balance between oxidation and the antioxidative defense system is known to play a role in spermatogenesis and fertility (3, 6–8). Other pathogenetic mechanisms thought to play a role in CP-induced testicular toxicity include decreased DNA synthesis in spermatogonia, decreased protein synthesis in spermatid, apoptosis and sperm dysfunction (5, 9). Many antioxidants and nutritional supplements have been used in experimental models to overcome CP-induced reproductive toxicity (3–5, 9), with most of these reporting encouraging results.

Amifostine was initially developed in order to protect people from the effects of nuclear weapons and was later developed as a cytoprotection for radiation therapy and chemotherapy (10). Amifostine (AMF; S-2-(3-aminopropylamino) ethylphosphothiotic acid) is known as a cytoprotective drug having antioxidant properties. It is a prodrug metabolized to an active form known as WR-1065 by interaction with membrane-bound alkaline phosphatase (ALP) (10, 11). WR-1065 is the metabolite responsible for the radical scavenging and antioxidant properties of amifostine (11).

Although amifostine has been shown to have no detrimental effects of doxorubicin on testis tissue (12, 13), a number of previous studies suggest that amifostine reduces seminiferous epithelium damage, with no improvement in fertility, in doxorubicin-treated rats (14), protects rats from cisplatin-induced testicular toxicity (15) and has a radioprotective effect on spermiogenetic cells when used prior to radiation (16).

The aim of the present study is to evaluate the possible beneficial effects of a cytoprotectant agent amifostine on testicular toxicity induced by CP, focusing on the cytoprotective effects of amifostine.

## 2. Materials and Methods

### Study animals and treatments

A total of 35 Sprague-Dawley rats weighing 200–250 gr were used in the study. They were housed in ventilated rooms at a temperature of 24 + 2°C with a 12 hr light/dark cycle, and a humidity of 60 + 5% and were fed with a standard pelleted diet with free access to water.

The rats were divided into four groups, each containing seven animals. The control group animals (C) received no intervention. The CP group animals received a single dose of 200 mg/kg CP on day 8 by intraperitoneal (IP) injection and were left untreated for the following seven days. The two remaining groups of animals (AMF 200 and AMF 400) were treated with 200 mg/kg/day amifostine and 400 mg/kg/day amifostine for seven days prior to and following a single IP injection of 200 mg/kg CP.

At the end of the treatment protocol, the rats were sacrificed by decapitation. The animals were then weighed with intracardiac blood samples obtained under ether anesthesia. After killing the animals with a high-dose ether, both testes were removed from each and weighed. Blood samples were centrifuged to obtain serum samples which were stored at 70°C until analysis. One of the testes was used for histological analysis and the other was weighed and stored at 70°C until the dry weight could be determined.

### Morphometrical evaluations

The body weights (BWs) of the animals were determined at the start of the study protocol. For a determination of the dry weight of testis, the samples stored at 70°C were dried in an oven at 60°C. The ratio of dry weight of testis (TW${}_{d}$) to its wet weight (TW${}_{w}$) was calculated as follows:

 TW d/w= TW d/ TW w.

Additionally, the ratio of TW${}_{w}$ to body weight (TW/BW) was also calculated to determine the relative testicular weight.

### Testis histology

Epididymis was removed with the testes and was used to evaluate sperm count and morphology. The epididymis was immediately put into 1 mL of Phosphate-buffered saline (PBS) and cut into small pieces. After vortexing for 2–3 min, 50 µL of supernatant was put on a Makler counting chamber (Sefi Medical Instruments, Haifa, Israel) to assess sperm count and morphology. Morphometric analysis was performed on all of the animals using image-analyzing software. Epithelial height was measured in seminiferous tubules at six areas for six experimental subjects per group.

Testicular tissue used for histological analysis was fixed in 4% paraformaldehyde and then embedded in paraffin blocks. Tissue sections cut at a thickness of 5 µm were stained with hematoxylin and eosin (H&E). The stained sections were evaluated under a light microscope. Tubuloseminiferous epithelial height was measured in order to determine the histological changes in H & E-stained preparations. A Johnsen scoring system was used to evaluate the germ cells in testicular tubules (17). Germ cells were scored from 1 to 10 according to their number and cell type of highest maturation in each tubule.

### Serum testosterone, LH, and FSH analyses

Serum testosterone, LH, and FSH levels were determined using commercially available kits for rats (USCN Life Science Inc., Wuhan, China).

### Ethical consideration

All animal experiments were conducted according to the guidelines for the care and use of laboratory animals with consent obtained from the local ethics committee (26/04/2011-205).

### Statistical analysis

The results are expressed as Mean ± SEM. A statistical analysis was performed using an SPSS 21.0 (Predictive Analytics, Chicago, IL) package program. The difference between groups was determined using a one-way ANOVA and a Kruskal-Wallis test. The statistical significance was set at p < 0.05.

## 3. Results

### Morphometrical evaluations

The BW of the animals did not differ among the study groups (p > 0.05). Accordingly, there was no significant difference in TW${}_{d/w}$ and TW/BW among the groups (p > 0.05) (Table I).

### Testis histology

**Figure 1:**
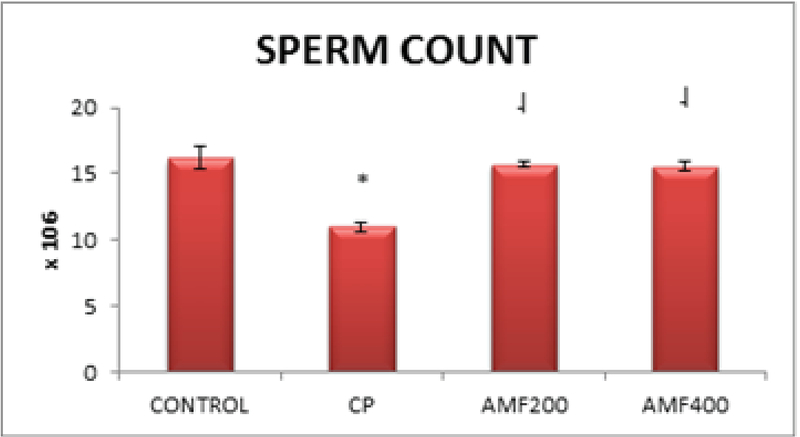
Epididymal sperm count. Differences are given compared to the control group (*) or CP group (
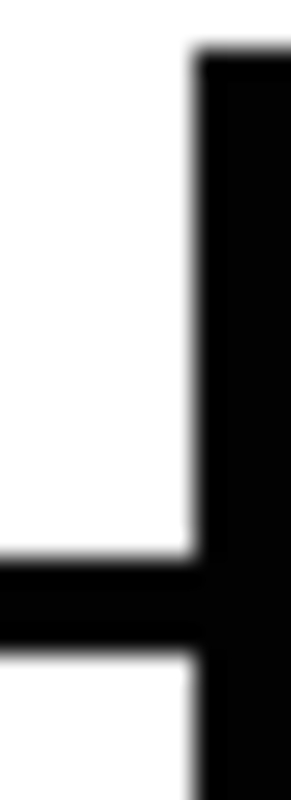
 ). CP: Cyclophosphamide group; AMF200 and AMF400: animal groups treated with 200 mg/kg/day and 400 mg/kg/day amifostine.

The epididymal sperm count was significantly lower in the CP-administered animals compared to the healthy control animals (p < 0.01). On the other hand, 200 mg/kg and 400 mg/kg amifostine resulted in a significantly higher sperm count compared to the CP group of animals (p < 0.01 and p < 0.05, respectively) (Figure 1).

**Figure 2:**
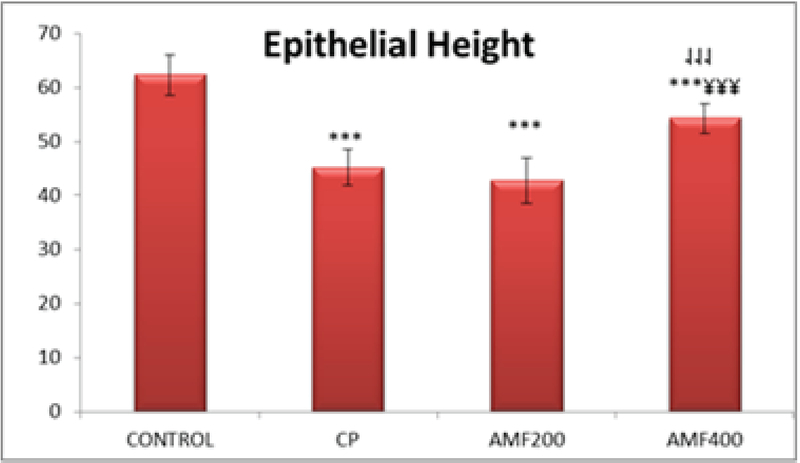
Testicular tubule epithelial height. Differences are given compared to the control group (*) or CP group (
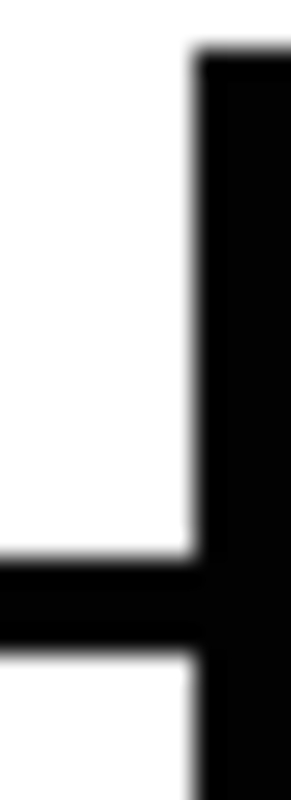
 ). CP: cyclophosphamide group and AMF200 group( ¥); AMF200 and AMF400: animal groups treated with 200 mg/kg/day and 400 mg/kg/day amifostine, p < 0.001.

The tubular epithelial height in the testis was significantly lower in the CP, AMF200, and AMF400 groups compared to the control animals (p < 0.001 for each). On the other hand, it was significantly higher in the AMF400 group compared to the CP and AMF200 groups (p < 0.001 for both) (Figure 2).

**Figure 3:**
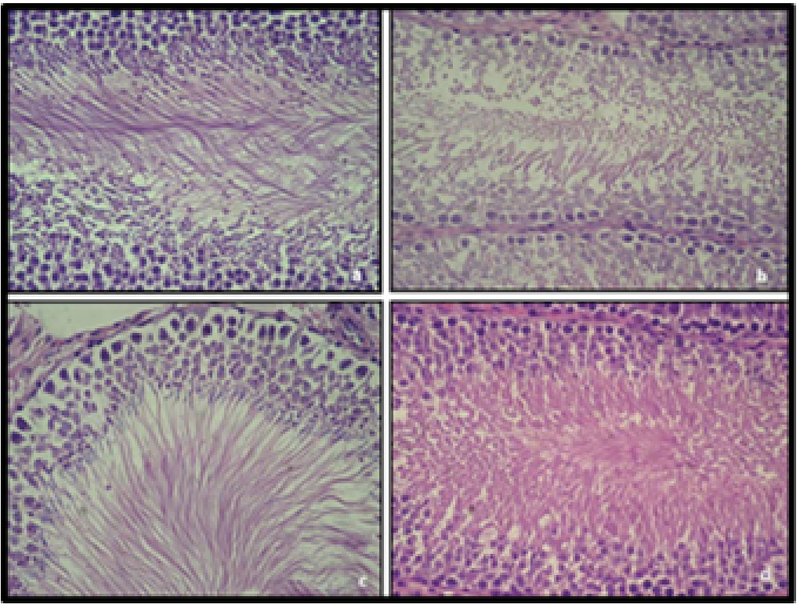
Histological analysis in the HE-stained testis preparations: (a) Control; (b) CP group; (c) AMF200 group; (d) AMF400 group.

The histological appearance of the testis is shown in Figure 3. H&E staining showed increased debris at the center of the tubules and damage in Sertoli cells in the CP and AMF200 groups. On the other hand, animals in the AMF400 group showed minimal debris in the tubules and no Sertoli cell damage (Figure 3).

**Figure 4:**
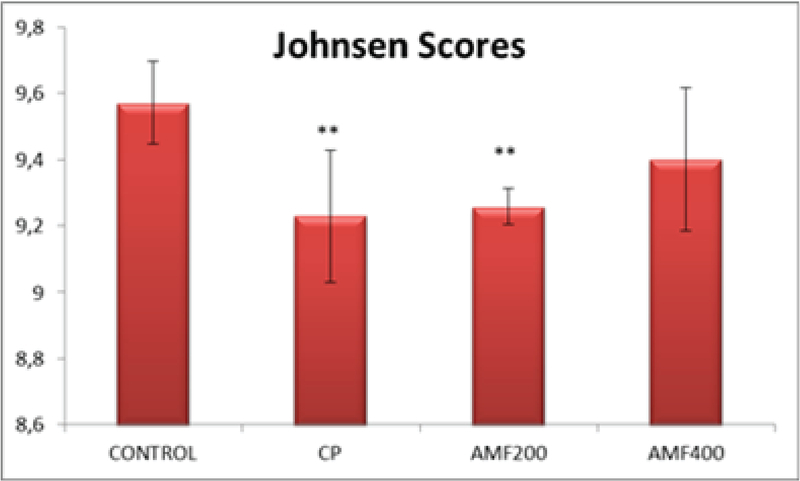
Johnsen scores. Differences are given compared to the control group (*); AMF200 and AMF400: animal groups treated with 200 mg/kg/day and 400 mg/kg/day amifostine p < 0.002.

Johnsen scores were found to be significantly decreased in the CP and AMF200 groups compared to the control animals (p > 0.01) (Figure 4). It should be noted that despite not being statistically significant, the scores were slightly higher in the AMF400 group.

### Serum testosterone, LH, and FSH analyses

**Figure 5:**
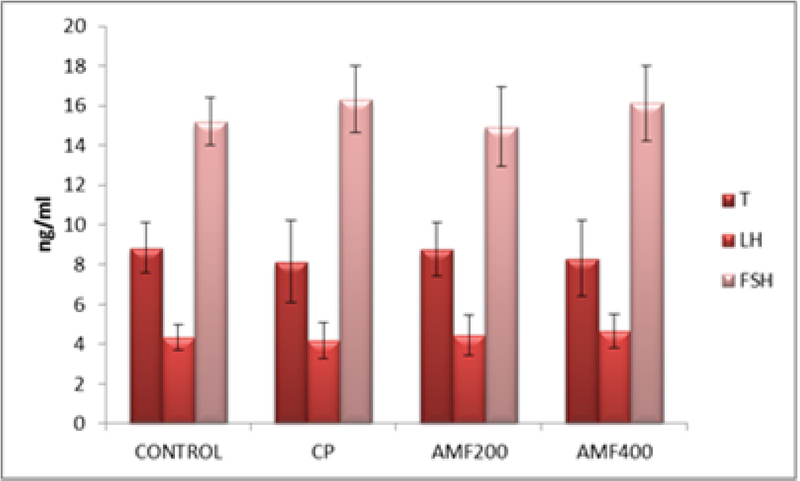
Serum testosterone, LH and FSH levels. CP: cyclophosphamide group; AMF200 and AMF400: animal groups treated with 200 mg/kg/day and 400 mg/kg/day amifostine.

The serum testosterone, LH and FSH levels measured by ELISA kits showed no difference among the groups (p > 0.05) (Figure 5).

**Table 1:** Body and organ weights in animals


	**Body weight (g)**	**TW${}_{d/w}$(mg/mg)**	**TW/BW (mg/g)**
CONTROL	202.9 ± 12.2	0.149 ± 0.40	0.0071 ± 0.003
CP	201.4 ± 13.5	0.165 ± 0.013	0.0063 ± 0.0005
AMF200	225.0 ± 26.9	0.171 ± 0.017	0.0055 ± 0.0004
AMF400	212.9 ± 29.8	0.175 ± 0.022	0.0057 ± 0.0003
Note: CP: Cyclophosphamide group; AMF200 and AMF400: animal groups treated with 200 mg/kg/day and 400 mg/kg/day amifostine; TW${}_{d/w}$: ratio of dry to wet weight of testis; TW/BW: ratio of testicular weight to body weight			

## 4. Discussion

Amifostine is a chemoprotective pro-drug transformed into its active form by ALP (18). Many previous studies have shown that amifostine may protect several tissues including bone marrow, peripheral nerves, heart, kidney, and salivary glands from the cytotoxic effects of an alkylating agent without compromising their antitumor activities (18).

The current study evaluates the protective effect of amifostine on testicular dysfunction induced by CP in male rats.

One remarkable finding in the present study is an increased sperm count in the amifostine-treated animals. The epididymal sperm count decreased significantly in animals treated only with CP, suggesting testicular dysfunction induced by CP (p < 0.01). However, amifostine, both in doses of 200 mg/kg/day and 400 mg/kg/day, significantly reversed this decrease in sperm count induced by CP (p < 0.01 and p < 0.05, respectively) (Figure 1). Nalca *et al* previously investigated the possible protective effect of amifostine on radiation-induced testicular damage. Similar to our findings, the authors reported an increased primary spermatocyte count with the amifostine treatment compared to radiation-treated animals (19), suggesting that amifostine may have the potential to protect the testis from damage.

Testicular epithelial height was significantly lower in the CP, AMF200, and AMF400 groups compared to the control group animals (p < 0.01) (Figure 2). It is not unexpected to have decreased epithelial height in animals treated with CP. The most remarkable finding was a significantly increased epithelial height in the AMF400 group compared to the CP group and even the AMF200 group. Epithelial height was also higher in the AMF200 group compared to the CP group with no statistical difference, suggesting that amifostine may protect the testicular structure against CP-induced damage in a dose-dependent manner. In a similarly designed study, Lirdi *et al* studied the effect of amifostine on testicular damage induced by cisplatin, a cytotoxic agent (15). The authors found similar results to those of our study and concluded that amifostine may protect testicular tissue from the adverse effects of cisplatin. On the other hand, Vendramini *et al* found that doxorubicin treatment decreases seminipherous epithelial height in prepubertal male rats which was prevented with the administration of a 400 mg/kg amifostine treatment (14). As can be seen in the aforementioned two studies, a 400 mg/kg amifostine treatment seems to have a protective effect on testicular tissue against chemotherapy-induced damage. Accordingly, amifostine was also effective at a dose of 400 mg/kg compared to a dose of 200 mg/kg. All of these results indicating that amifostine may protect testicular tissue from damage suggest that amifostine could be further studied for use as a chemoprotective agent, particularly in young males.

All histological findings also support the abovementioned findings (Figure 3). Decreased debris in the center of tubules suggests that 400 mg/kg amifostine might protect the CP-induced testicular damage. Johnsen scores were found to be significantly lower in the CP and AMF200 groups (Figure 4). Although not statistically significant, the scores were higher in the AMF400 group, suggesting a dose-dependent increase for amifostine in the Johnsen scores.

There were no significant differences in the testosterone, LH and FSH levels among the groups (Figure 5). Jahnukainen *et al* studied the protective effect of amifostine on the testicular tissue of immature rats and also found no significant difference between the groups in serum testosterone levels (12). Therefore, the authors concluded that amifostine may not have a protective effect on testis. Although our serum hormone levels are similar to those observed by Jahnukainen *et al*, our histological findings suggest a marked improvement in testicular tissue (12). Accordingly, Lirdi *et al* found that amifostine preserved the seminipherous epithelium of male rats from the deleterious effects of cisplatin and conclude that amifostin may be a beneficial option for infant and young male patients treated with chemotherapeutic agents (15).

Although there have been many studies on the potential protective effects of amifostine from chemotherapy-induced adverse effects on several tissues, to our knowledge, there has been no in vivo study investigating the effect of amifostine on CP-induced testicular toxicity. In a germ cell study, BirGolkar-Narenji *et al* investigated the protective effect of amifostine on damage induced by CP in mice oocytes (20). The authors suggest that amifostine may protect the mice oocytes from apoptosis induced by CP, supporting our in vivo results.

## 5. Conclusion

Cyclophosphamide has been suggested that CP results in germ cell damage in both males and females (21). Amifostine, approved as a chemoprotective agent by the FDA, is known to have cytoprotective effects. It acts as a free radical scavenger and ameliorates the effects of ionizing radiation and DNA damage (22). The current study on the possible protective effects of amifostine on testicular toxicity induced by CP in rats suggests that amifostine, at a dose of 400 mg/kg, may have a protective effect on testicular damage. This is of importance for male patients undergoing cancer treatment wishing to maintain reproductive ability.

##  Conflict of Interest

The authors report no conflict of interest.
